# Crystal structure of the multidrug resistance regulator RamR complexed with bile acids

**DOI:** 10.1038/s41598-018-36025-8

**Published:** 2019-01-17

**Authors:** Suguru Yamasaki, Ryosuke Nakashima, Keisuke Sakurai, Sylvie Baucheron, Etienne Giraud, Benoît Doublet, Axel Cloeckaert, Kunihiko Nishino

**Affiliations:** 1 Graduate School of Pharmaceutical Sciences, Osaka University, 1-6 Yamadaoka, Suita, 565-0871 Osaka Japan; 2 Institute of Scientific and Industrial Research, Osaka University, 8-1 Mihogaoka, Ibaraki, Osaka 567-0047 Japan; 3 INRA, UMR1282 Infectiologie et Santé Publique, F-37380 Nouzilly, France; 4 Université François Rabelais de Tours, UMR1282 Infectiologie et Santé Publique, F-37000 Tours, France

## Abstract

During infection, *Salmonella* senses and responds to harsh environments within the host. Persistence in a bile-rich environment is important for *Salmonella* to infect the small intestine or gallbladder and the multidrug efflux system AcrAB-TolC is required for bile resistance. The genes encoding this system are mainly regulated by the *ramRA* locus, which is composed of the divergently transcribed *ramA* and *ramR* genes. The *acrAB* and *tolC* genes are transcriptionally activated by RamA, whose encoding gene is itself transcriptionally repressed by RamR. RamR recognizes multiple drugs; however, the identity of the environmental signals to which it responds is unclear. Here, we describe the crystal structures of RamR in complexes with bile components, including cholic acid and chenodeoxycholic acid, determined at resolutions of 2.0 and 1.8 Å, respectively. Both cholic and chenodeoxycholic acids form four hydrogen bonds with Tyr59, Thr85, Ser137 and Asp152 of RamR, instead of π–π interactions with Phe155, a residue that is important for the recognition of multiple compounds including berberine, crystal violet, dequalinium, ethidium bromide and rhodamine 6 G. Binding of these compounds to RamR reduces its DNA-binding affinity, resulting in the increased transcription of *ramA* and *acrAB-tolC*. Our results reveal that *Salmonella* senses bile acid components through RamR and then upregulates the expression of RamA, which can lead to induction of *acrAB-tolC* expression with resulting tolerance to bile-rich environments.

## Introduction

*Salmonella* is a bacterial pathogen that causes a variety of foodborne illnesses in humans. During the course of its infection of the intestinal tract and gallbladder, *Salmonella* is exposed to bile acids. These are detergent-like biological substances that are synthesized in the liver from cholesterol and stored in the gallbladder. These bile acids possess strong antimicrobial activity, disrupt cell membranes, denature proteins and trigger DNA damage^1,2^. Enteric bacteria such as *Salmonella* must tolerate the presence of bile acids in order to survive in the gastrointestinal transit and gallbladder^3,4^. Enteric bacteria have developed an intrinsic resistance to the toxic effects of bile acids, due to the low permeability of their outer membranes to lipophilic solutes and the presence of active efflux mechanisms^5,6^.

**Figure 1:**
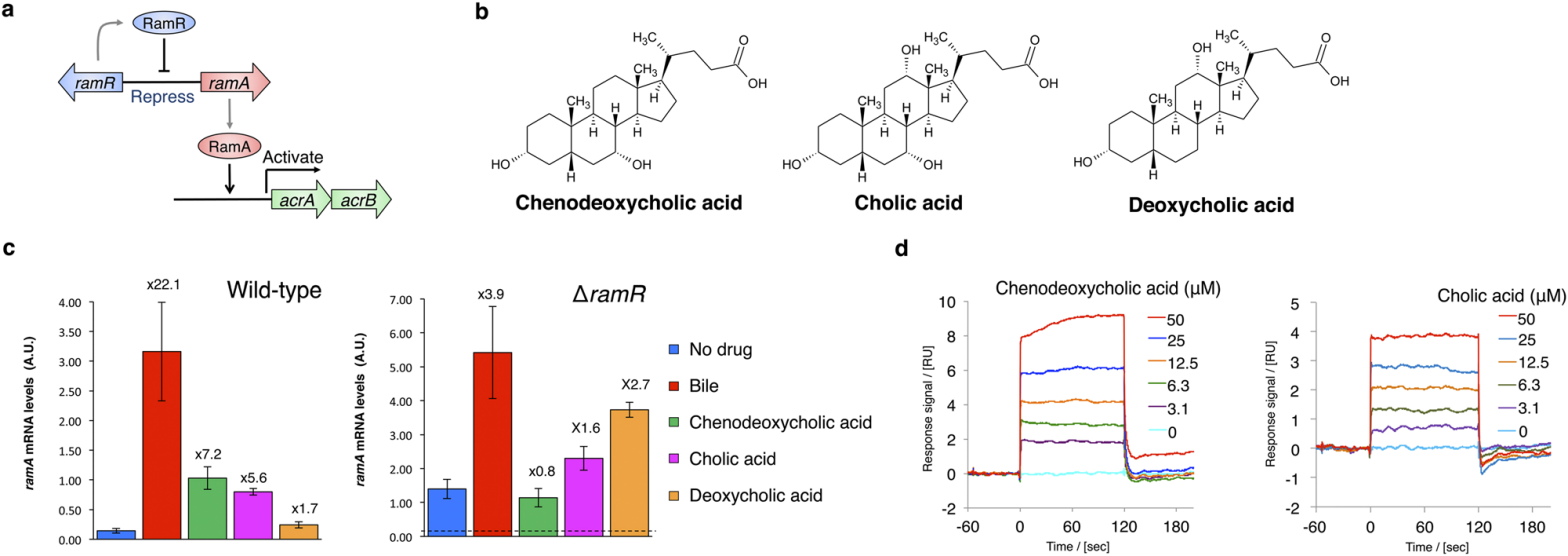
Chenodeoxycholic and cholic acids activate the *ramA* gene in a RamR-dependent manner. (**a**) Model for gene regulation pathway by RamR. RamR represses the *ramA* gene encoding the activator protein for the *acrAB* drug efflux pump genes. RamR binds to the region between the *ramR* and *ramA* genes, while RamA binds to the upstream region to *acrAB*. (**b**) The chemical structures of chenodeoxycholic, cholic and deoxycholic acids. (**c**) Effect of bile and chenodeoxycholic, cholic and deoxycholic acids on the expression of *ramA* in wild-type and ∆*ramR Salmonella* strains, as assessed by qRT-PCR. Cells were grown in LB broth supplemented with 25.6 mg/ml bile, or 5 mM of chenodeoxycholic, cholic or deoxycholic acids. Values above the bars indicate the fold difference in *ramA* mRNA levels relative to the control in the same strain (wild-type or ∆*ramR)*. In the right panel (expression in ∆*ramR)*, the horizontal broken lines represents *ramA* mRNA levels in the wild-type control. The error bars indicate the standard deviation from three independent replicates. A.U., arbitrary units. (**d**) Binding of chenodeoxycholic and cholic acids to RamR detected by SPR analysis. RamR was immobilized onto a sensor chip and then the chenodeoxycholic or cholic acid was passed over the sensor surface at the various concentrations indicated. A representative result from one of three experiments that produced similar data is shown. SPR, surface plasmon resonance.

*Salmonella enterica* serovar Typhimurium (*S*. Typhimurium) contains at least nine multidrug efflux systems^7^. Among these, the AcrAB-TolC system, whose AcrB transporter belongs to the resistance–nodulation–cell division family, is particularly effective in generating bile acid resistance^7,8^. Bile induces the expression of *acrAB*^8^, and this induction is mediated by the transcriptional regulators RamA and RamR^9,10^. The global transcriptional activator RamA, which belongs to the AraC/XylS family of regulatory proteins, activates the expression of *acrAB* and *tolC* genes^9^. The local transcriptional repressor RamR, which is located directly upstream of *ramA*, belongs to the TetR family of regulatory proteins and represses the expression of both *ramA* and *ramR* (Fig. 1a)^10,11^. Additionally, it has recently been found that bile inhibits the binding of RamR to the *ramA* promoter region and activates *ramA* gene expression, resulting in the increased expression of *acrAB* and *tolC*^10^.

Our previous biochemical and structural studies found that RamR can recognize multiple compounds, including berberine, crystal violet, dequalinium, ethidium bromide and rhodamine 6 G^12^. Binding of these compounds to RamR results in the increased expression of *ramA*; all the compounds recognized by RamR are also known substrates of AcrAB. However, these five drug compounds are not usually present in environments inhabited by *Salmonella*, such as the intestine, and so, because bile induces *ramA* expression, we hypothesized that RamR also recognizes some components of bile acids^10^.

There are several derivatives of bile acids. Two primary compounds, cholic (3α, 7α, 12α-trihydroxy-5β-cholan-24-oic acid; Fig. 1b) and chenodeoxycholic acids (3α, 7α-dihydroxy-5β-cholan-24-oic acid; Fig. 1b) belong to the C24 group, whose members have steroidal backbones and are metabolized from cholesterol in hepatocytes. Primary bile acids are metabolized in the liver, via conjugation to glycine or taurine^13^. In the intestine, a broad range of intestinal anaerobic bacteria modify primary bile acids through hydrolysis and hydroxyl group dehydrogenation to produce secondary bile acids, such as deoxycholic acid (3α, 12αdihydroxy-5β-cholan-24-oic acid; Fig. 1b) and lithocholic acid (3α-hydroxy-5β-cholan-24-oic acid). Here, we report the crystal structure of RamR in complex with cholic and chenodeoxycholic acids. Both cholic and chenodeoxycholic acids decreased the DNA-binding affinity of RamR, resulting in increased transcription of *ramA*.

## Results

### Cholic and chenodeoxycholic acids induce *ramA* expression in a RamR-dependent manner

It has previously been shown that *ramA* expression is activated by a crude ox-bile extract, which is mainly dependent on RamR and is required for the bile-mediated transcriptional activation of *acrAB* and *tolC* genes^10^. However, the components of bile that influence the expression level of *ramA* have, to date, remained unknown. To identify these components and investigate their possible action on RamR, we tested the effect of a crude ox-bile extract, primary bile acids (cholic and chenodeoxycholic acids), and a secondary bile acid (deoxycholic acid) on the expression level of *ramA* in the wild-type *S*. Typhimurium strain and its *ramR* deletion mutant (Fig. 1c). We confirmed that the bile extract increased the *ramA* transcript level by approximately 20-fold in the wild-type strain, but only by 3.9-fold in the *ramR* deletion mutant (Fig. 1c). Among the bile acid components tested, chenodeoxycholic and cholic acids increased *ramA* expression by more than 5.0-fold in the wild-type strain (7.2- and 5.6-fold, respectively; Fig. 1c), but not in the mutant (a 0.8-fold reduction and a 1.6-fold increase, respectively; Fig. 1c). These results indicate that *ramA* induction by cholic and chenodeoxycholic acids is dependent on RamR. In contrast, deoxycholic acid increased *ramA* expression only slightly; this effect appeared to be independent of RamR, as it was similar in the wild-type strain (1.7-fold increase) and in the *ramR* deletion mutant (2.7-fold increase).

### Binding of bile acids to RamR

Because cholic and chenodeoxycholic acids induce *ramA* in a RamR-dependent manner, we hypothesized that these compounds are recognized by RamR. To test the possibility that cholic and chenodeoxycholic acids may bind to RamR, we employed surface plasmon resonance (SPR) analysis using a Biacore T200 instrument (GE Healthcare). Cholic and chenodeoxycholic acids were separately passed over RamR immobilized on a CM5 sensor chip and the SPR responses indicated that both bound directly to RamR, with the responses increasing in a concentration-dependent manner (Fig. 1d). The *K*_D_ values obtained from SPR data for chenodeoxycholic and cholic acids were 65.3 µM and 60.5 µM, respectively.

### Co-crystal structures of RamR with cholic acid and chenodeoxycholic acid

**Figure 2:**
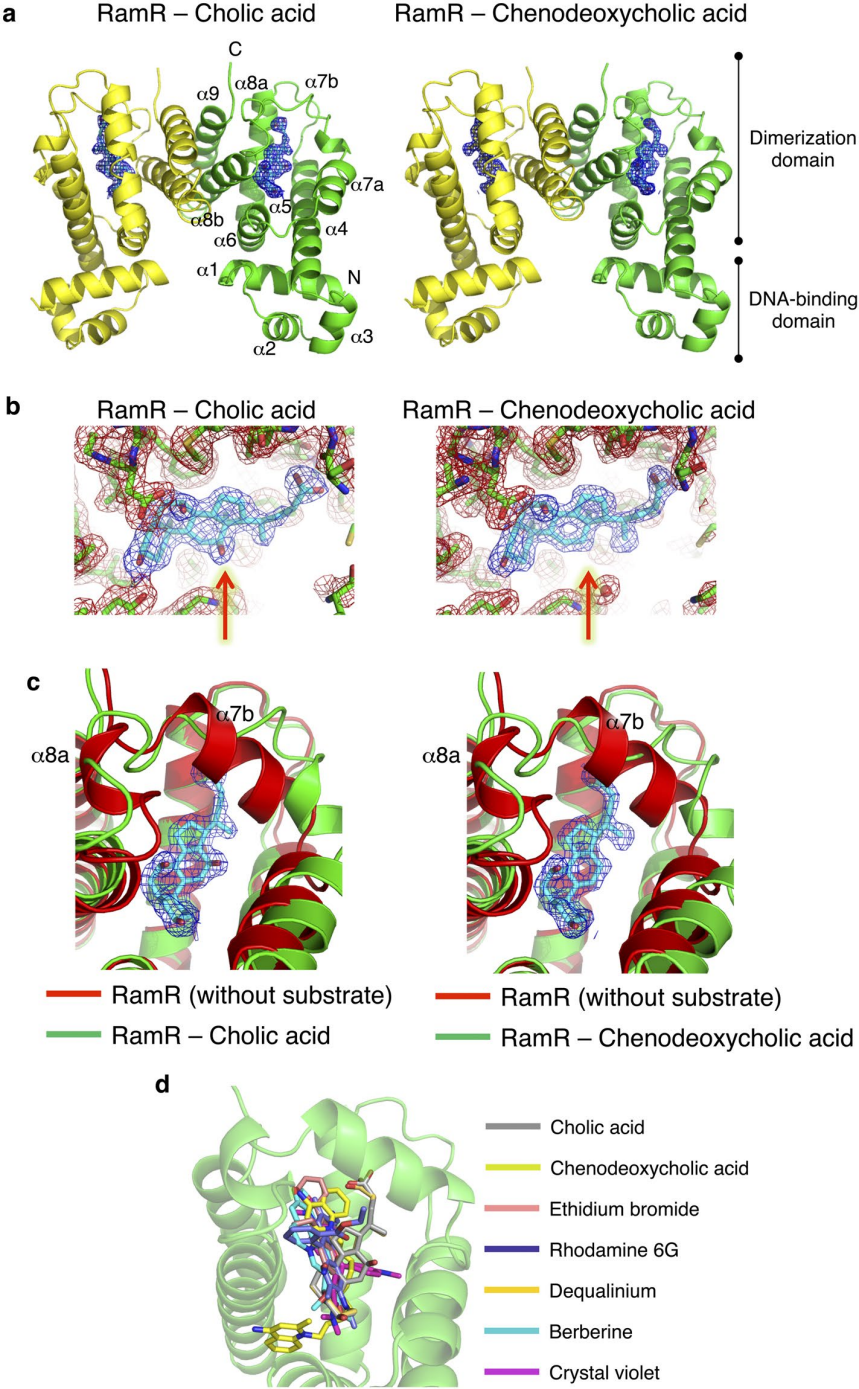
Co-crystal structures of RamR with cholic and chenodeoxycholic acids. (**a**) Full structure of the RamR dimer bound to two molecules of cholic or chenodeoxycholic acid. The α helices in RamR are indicated as α1, α2, α3, α4, α5, α6, α7a, α7b, α8a, α8b and α9. α7b and α8a are uncoiled upon the binding of cholic or chenodeoxycholic acid. 2*F*_o_ − *F*_c_ electron densities for cholic and chenodeoxycholic acids are show as a blue mesh contoured at 1.2*σ*. The carbon atoms of cholic or chenodeoxycholic acid are shown in cyan and oxygen atoms are shown in red. (**b**) Close-up view of the ligand binding site. Electron density map of the protein moiety (red mesh) and cholic and chenodeoxycholic acids (blue mesh) are contoured at 1.2*σ*. Carbon atoms of bile acid components and RamR are shown in cyan and green, respectively. Nitrogen, oxygen and sulfur atoms are shown in blue, red and yellow, respectively. The presence of a 12-hydroxyl group in cholic acid and its absence in chenodeoxycholic acid are indicated by red arrows. (**c**) Comparison of the unligated RamR structure (indicated as a red ribbon, PDB ID: 3VVX) with the ligated (cholic acid: 6IE8 or chenodeoxycholic acid: 6IE9) structures (indicated as green ribbons). 2*F*_o_ − *F*_c_ electron density for cholic acid and chenodeoxycholic acid is shown as a blue mesh, contoured at 1.2*σ*. Carbon atoms of cholic or chenodeoxycholic acids are shown in cyan, and oxygen atoms are shown in red. Superimposed structures indicate that binding of cholic acid or chenodeoxycholic acid triggers uncoiling of helices α7b and α8a. (**d**) The superposition of the RamR ligands determined in previous studies and this study. Carbon atoms of cholic acid (PDB ID: 6IE8), chenodeoxycholic acid (6IE9), ethidium bromide (3VVY), rhodamine 6G (3VVZ), dequalinium (3VW0), berberine (3VW2) and crystal violet (3VW1) are shown in gray, light green, pink, blue, yellow, light blue and purple, respectively. Other objects are colored as in (**b**). The imposed image indicates that the carboxyl groups of cholic and chenodeoxycholic acids are extended in the direction of the α7b helix. The binding locations of cholic and chenodeoxycholic acids are different from those observed for the five antimicrobial compounds.

**Figure 3:**
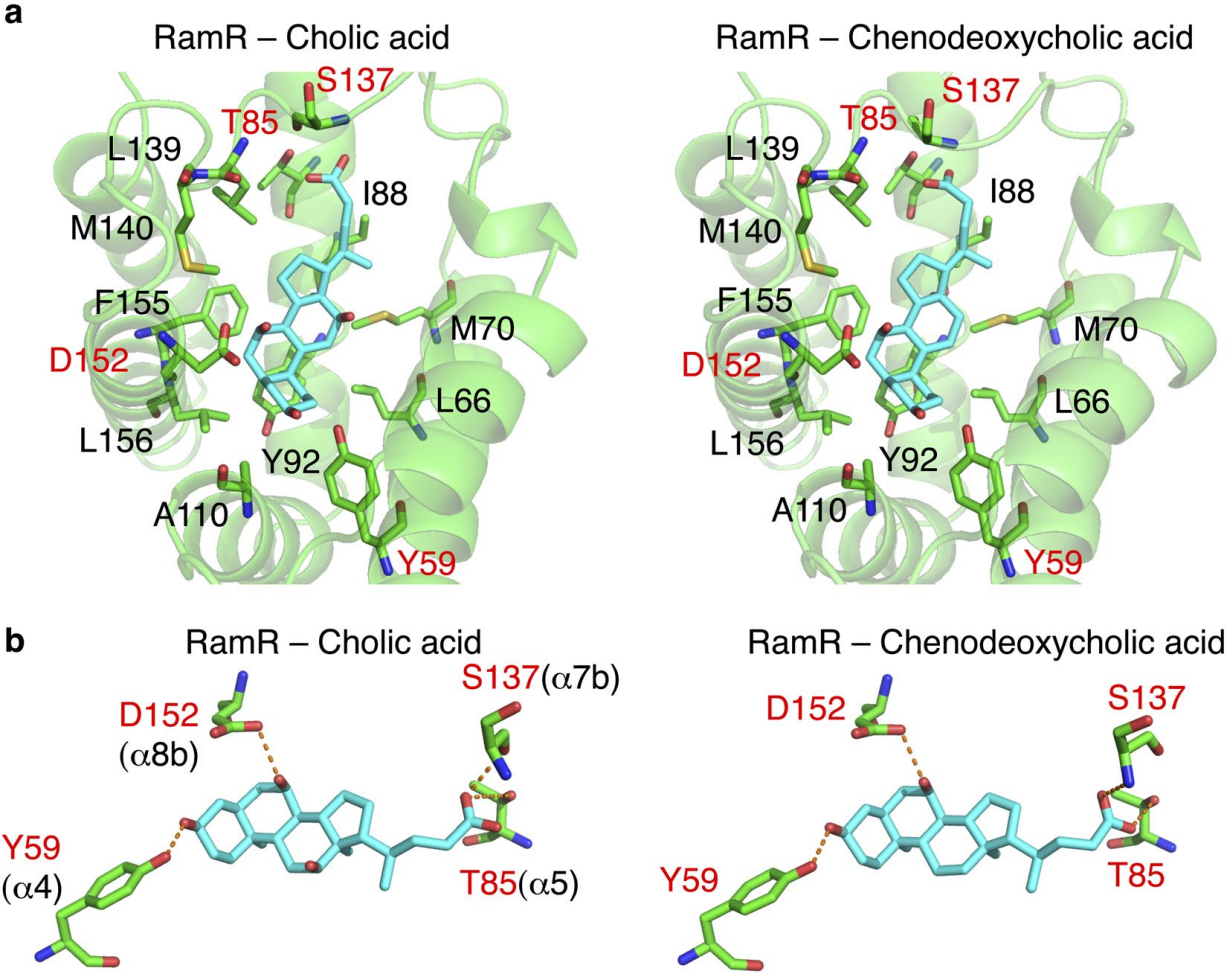
Recognition of bile acids by RamR. (**a**) The substrate-binding site of RamR with bound cholic and chenodeoxycholic acid molecules. Key residues that are involved in forming hydrogen bonds with cholic or chenodeoxycholic acid are shown, including Tyr59, Thr85, Ser137 and Asp152 (indicated by red characters). Objects are colored as in Fig. 2. (**b**) Close-up view of the substrate-binding site of RamR containing cholic acid or chenodeoxycholic acid. Objects are colored as in Fig. 2, and key residues are colored as in Fig. 2a. Hydrogen bonds are indicated by dotted red lines.

To elucidate the recognition mechanism of cholic and chenodeoxycholic acids by RamR, we determined the individual crystal structures of RamR complexed to both of these compounds (Fig. 2a). Electron density maps of each co-crystal structure are shown in Fig. 2b and these structures were refined to resolutions of 2.0 and 1.8 Å for cholic and chenodeoxycholic acids, respectively (Supplementary Table S1). As shown in Fig. 2a, RamR binds two molecules of either cholic or chenodeoxycholic acid per dimer. Both compounds were completely enclosed by the RamR-binding pockets. The structural difference between cholic and chenodeoxycholic acid lies in the presence or absence of a 12-hydroxyl group (Fig. 1b), which in turn results in a difference in the electron density maps of RamR bound to these compounds (Fig. 2b, indicated by red arrows). Comparison of the ligated structures with unligated RamR revealed that the binding of cholic or chenodeoxycholic acid triggers uncoiling of the α7b and α8a helices (Fig. 2c), suggesting that RamR recognizes these compounds via an induced-fit mechanism. The binding position of cholic or chenodeoxycholic acid collides with helix α7b (indicated by a red ribbon in Fig. 2c) of the unliganded RamR. Thus, the uncoiling of α7b of RamR (indicated as a green ribbon in Fig. 2c) is required for the binding of these bile acid components. The α8a helix is also uncoiled in ligated RamR, probably because of the uncoiling of α7b. These uncoiled forms of α7b and α8a were not observed in RamR structures bound to multiple other drugs (Fig. 2d)^12^, indicating that this helix uncoiling is required for the recognition of bile acids. Although the five antimicrobial drugs were found to form π–π interactions with Phe155 in a previous RamR structural study, this interaction does not occur with cholic or chenodeoxycholic acids (Fig. 3a and Supplementary Table S2). Instead of a π–π interaction, both bile acids form four hydrogen bonds with the Tyr59, Thr85, Ser137 and Asp152 residues of RamR (Fig. 3b). This indicates that both cholic and chenodeoxycholic acids are recognized by RamR via the same mechanism. These interactions are different from the hydrogen bonds formed by the five antimicrobial drugs with other amino acid residues of RamR (Supplementary Table S2), indicating that the mechanism of bile acid recognition is separate to those of other antimicrobial drugs. The interaction of different sets of amino acid residues with each compound indicates that multiple compounds are recognized by the multisite-binding pocket of RamR.

### Reduction of RamR DNA-binding affinity by cholic acid and chenodeoxycholic acid

**Figure 4:**
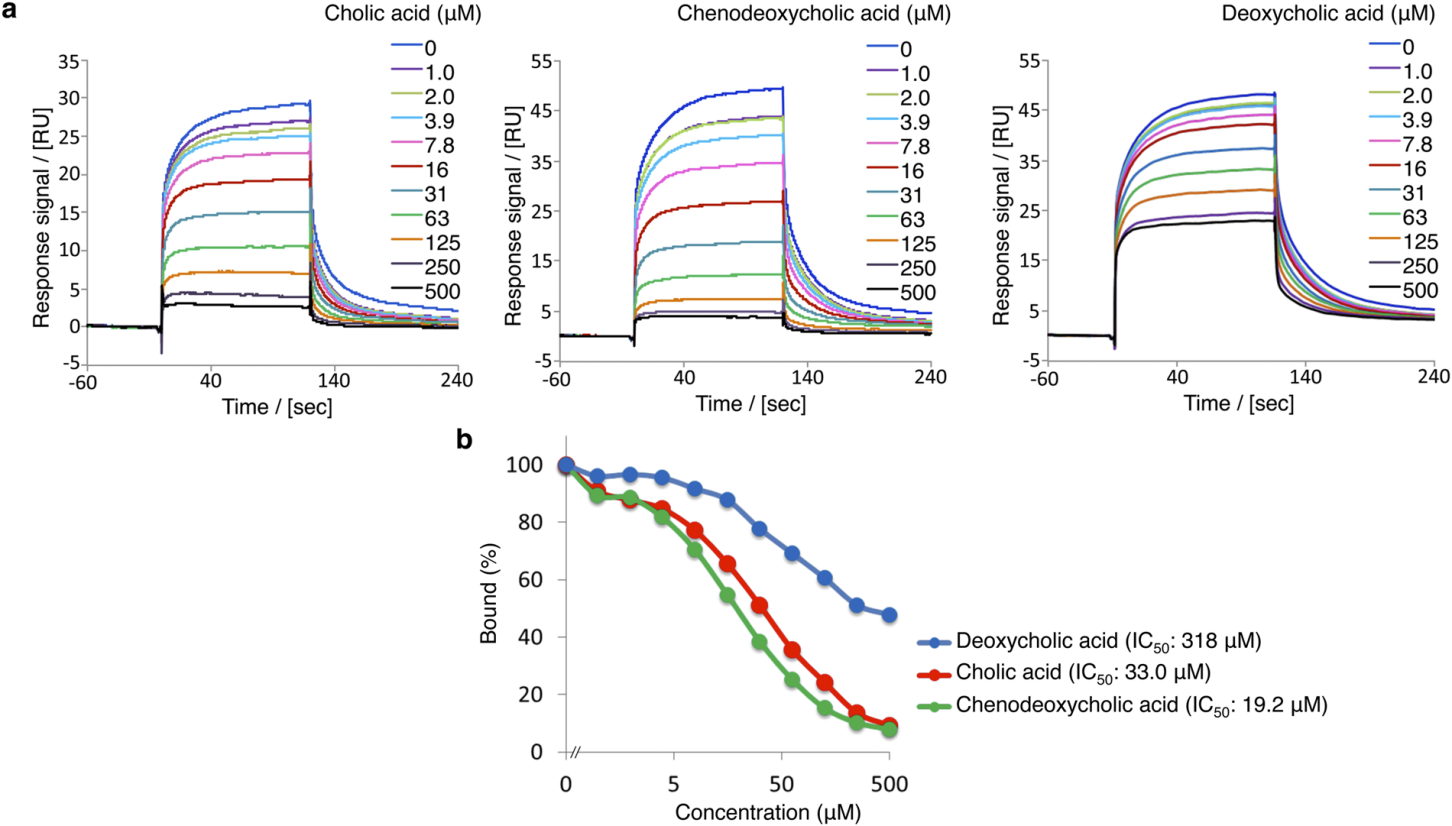
Inhibitory effects of bile acids on the DNA affinity of RamR. (**a**) Inhibitory effects of cholic or chenodeoxycholic acid on the DNA-binding affinity of RamR analyzed by SPR. DNA was immobilized onto a sensor chip and purified RamR protein was passed over the sensor surface in the presence or absence of bile acids at the various concentrations indicated. (**b**) Inhibitory effects were calculated from the results of SPR analysis. Percentage binding was calculated relative to the amount of RamR-binding to DNA in the absence of bile acids, which was assigned as 100%. The result of one of three experiments that produced similar data is shown. SPR, surface plasmon resonance.

Baucheron *et al*. proposed that RamR interacts with P_*ramA*_ as a dimer of dimers^14^. We previously showed that the binding of multiple compounds to RamR reduces its DNA-binding affinity and results in the induction of *ramA* expression^12^. The results of the present study suggest that the binding of cholic acid or chenodeoxycholic acid to RamR may also reduce its DNA-binding affinity. To quantify the effects of cholic and chenodeoxycholic acids on the interaction of RamR with its DNA-binding site, we performed SPR experiments using a purified RamR protein and 100-bp DNA fragment containing the RamR-binding site. RamR protein solution was passed over the DNA fragment immobilized on a sensor chip while in the presence or absence of cholic or chenodeoxycholic acid (up to 500 µM). Both bile acids inhibited the binding of RamR to the DNA, in a concentration-dependent manner (Fig. 4). The IC_50_ values of cholic and chenodeoxycholic acids derived from the SPR data were 33.0 µM and 19.2 µM, respectively. In contrast, the much higher IC_50_ value obtained with deoxycholic acid (318 µM) indicated a much weaker ability of this bile acid to reduce the RamR DNA-binding affinity (Fig. 4). This result is in good agreement with the qRT-PCR data, showing a lower activation of *ramA* expression by deoxycholic acid than by cholic and chenodeoxycholic acids (Fig. 1c). Collectively, these results demonstrate that the interaction of cholic and chenodeoxycholic acids with RamR reduce its DNA-binding affinity and induce *ramA* expression.

## Discussion

RamA is known to be a major activator protein of the AcrAB-TolC efflux system in *S*. Typhimurium, thus, it is involved in resistance to multiple drugs. Here, we report a regulatory mechanism for the expression of *ramA* that involves the binding of its local RamR repressor protein to bile acids. The DNA-binding activity of RamR appears to be controlled by major bile acids, such as cholic or chenodeoxycholic acids.

The transcription of *acrAB* and *tolC* in *Salmonella* was previously known to be activated by bile^8^. However, the regulatory mechanisms involved in this activation have not been reported. The activation of *acrAB* in *Salmonella* in response to bile seems to be independent of other regulatory proteins, such as MarRAB, Rob, RpoS or PhoP-PhoQ^8^. Since the AcrAB-TolC efflux system exports bile salts and functions in both the bile resistance^15^ and pathogenesis of *Salmonella*^7,16,17^, it is important to understand the regulatory mechanism(s) of the *acrAB* and *tolC* genes in response to bile. We previously reported that bile induces AcrAB efflux pump expression in *Salmonella* through a specific regulatory protein, RamA, and, additionally, that cholic acid appears able to bind to this protein^9^. More recently, we also identified a different induction mechanism of *acrAB* in response to bile, whereby the bile-mediated activation of the *acrAB* and *tolC* multidrug efflux genes occurs via transcriptional derepression of the *ramA* activator gene, likely via the RamR repressor protein controlling expression of *ramA*^10^. Although the relative importance of both mechanisms has not yet been investigated, we suspect that these two different modes of regulation may coexist. A possible scenario is that bile first binds to and activates RamA and then, when the concentration of bile increases, proceeds to bind to RamR and stimulates RamA expression and the subsequent overproduction of the AcrAB-TolC efflux system. This scenario, however, requires further careful investigation.

The crystal structures of RamR in complexes with the components of bile reveal that both cholic and chenodeoxycholic acids form four hydrogen bonds with the Tyr59, Thr85, Ser137 and Asp152 residues of RamR, instead of π–π interaction with Phe155, an important residue for the recognition of multiple other drugs. Both cholic and chenodeoxycholic acids, but not deoxycholic acid, induce *ramA* expression in a RamR-dependent manner. We tried to crystalize RamR bound to deoxycholic acid but failed, probably because it lacks the 7α-hydroxyl group that is important in forming a hydrogen bond with Asp152 of RamR. Indeed, the inhibitory effect of deoxycholic acid on RamR-binding to the *ramA* promoter region is much weaker than that of cholic and chenodeoxycholic acids (Fig. 4). These acids inhibit interaction between RamR and the DNA fragment by approximately 90% or more at a concentration of 500 µM, whereas deoxycholic acid inhibits DNA binding by approximately 50% at the same concentration (Fig. 4b). This is consistent with the expression data, which show that deoxycholic acid induces *ramA* much less than cholic and chenodeoxycholic acids.

A similar approach to crystallizing a TetR-family regulator with bile acids has also been reported for CmeR^18^, and its structures complexed with taurocholic or cholic acids have been determined at resolutions of 2.2 and 2.4 Å, respectively. These two elongated bile acids did not bind in the same orientation inside the CmeR tunnel but, in fact, lay antiparallel to each other. The crystal structure of CmeR complexed with glycerol suggests the presence of at least two distinct binding sites^19^, while the CmeR-bile acid structures indicated that the large taurocholic and cholic acid molecules did not span the predicted binding sites, but instead bound to a distinct second site and left the glycerol-binding site unoccupied^18^. Unlike the bile acid-binding site in CmeR, the RamR-binding site of cholic and chenodeoxycholic acids is located on the upper side of the protein at almost the same position as that of the five antimicrobial drugs berberine, crystal violet, dequalinium, ethidium bromide and rhodamine 6 G (Fig. 2d). The α7b and α8a helices were uncoiled in the structures of RamR in complex with cholic and chenodeoxycholic acids, but such uncoiling was not observed in RamR bound to the five antimicrobial drugs or CmeR in complex with taurocholic or cholic acids. Uncoiling of the α7b and α8a helices probably occurred due to the smaller size of the substrate-binding site of RamR compared to the relatively large cholic and chenodeoxycholic acid molecules.

In conclusion, we have extended our knowledge of the recognition of bile acids by RamR, a regulator of multidrug resistance in several enterobacterial pathogens^20^. Cholic and chenodeoxycholic acids both form four hydrogen bounds with RamR, instead of the π–π interaction that is important for recognition of other drugs. These different recognition mechanisms highlight the wide substrate specificity of RamR, whereby the substrate-binding pocket accommodates a diverse array of ligands.

## Methods

### Bacterial strains, plasmids and growth conditions

The bacterial strains and the plasmids used in this study are listed in Supplementary Table 3. The *S. enterica* serovar Typhimurium strains were the wild-type strain ATCC14028s^21^ and its ∆*ramR* mutant (which was produced as described previously)^22^. Bacterial strains were grown at 37 °C in Luria–Bertani broth supplemented, when appropriate, with ampicillin (100 µg/ml), kanamycin (25 µg/ml) or chloramphenicol (25 µg/ml).

In order to investigate the effects of the bile acids on *ramA* expression, 25.6 mg/ml of a crude ox bile extract purchased under the label ‘sodium choleate’ (Sigma-Aldrich, S9875), or 5 mM sodium cholate hydrate, sodium chenodeoxycholate or sodium deoxycholate monohydrate (C6445, C8261 and D5670, respectively, and all from Sigma) was added to the medium. Sodium choleate, whose precise composition was not determined, has previously been used in *Salmonella* gene expression experiments at concentrations of 3% (w/v), i.e., 30 mg/mL or higher^8^. We chose a concentration of 25.6 mg/mL, because it was the highest concentration that allowed the normal growth of the *Salmonella* strains used in this study.

### Gene expression analysis by qRT-PCR

Bacteria were grown until mid-log phase (OD_600_ of 0.6) and harvested by centrifugation. Pelleted cells were stabilized with RNAprotect Bacteria Reagent (Qiagen) and stored at −80 °C until needed. Total RNA was extracted using an RNeasy Mini kit (Qiagen). Removal of residual genomic DNA was performed using a Turbo DNA-free kit (Ambion) and checked by negative PCR amplification of the chromosomal sequence. RNA integrity was verified by electrophoresis on a 1% agarose gel. Total RNA (1.5 µg) was reverse-transcribed using random hexamers and the Superscript III First Strand Synthesis System (Applied Biosystems). The expression level of each gene of interest was calculated as the average of the results from three independent cDNA samples. For each cDNA sample and each gene, qRT-PCR runs were performed in duplicated wells and the primers used for qRT-PCR are listed in Supplementary Table 4. The cycling conditions were: 95 °C for 5 min followed by 40 cycles of 95 °C for 10 s and then 60 °C for 15 s. After each run, amplification specificity and the absence of primer dimers were verified using a dissociation curve, acquired by heating the PCR products from 60 °C to 95 °C. The relative quantities of transcripts were determined using a standard curve and normalized against the geometric mean of three reference genes (*gmk*, *gyrB* and *rrs*). A two-tailed Student’s *t* test was used to assess significance using a *p* value < 0.05 as cutoff.

### Crystallization, data collection, and structure determination

Purified RamR protein was prepared as described previously^12^. Co-crystals of RamR with cholic and chenodeoxycholic acids were grown from hanging drops at 25 °C using the vapor diffusion method. To form co-crystals, a 5-fold molar excess of cholic or chenodeoxycholic acids were added to 20 mg/ml RamR, then incubated overnight. The protein solution contained 20 mM sodium phosphate (pH 6.6), 75 mM NaCl and 2 mM DTT. The crystals grew to optimal size within 1 week and were cryoprotected using a solution containing 20% glycerol. Crystals were picked using LithoLoops (Protein Wave) and subjected to flash cooling in a cold nitrogen gas stream (100 K) from a cryostat (Oxford Cryosystems). All data sets were collected on beamline BL44XU at SPring-8 with a CCD detector MX225-HE (Rayonix) and at a cryogenic temperature of 100 K. The diffraction data were processed and scaled using the HKL2000^23^ package. The crystal structures of bile-bound RamR were determined at 2.00 and 1.78 Å resolution by the molecular replacement method using the program MOLREP^24^ from the CCP4 software suite. The atomic coordinates of the ligand-free RamR (Protein Data Bank [PDB] code: 3VVX) were used as the search model. The model was refined with the CNS^25^, REFMAC^26^ and COOT^27^ software and stereochemical quality was assessed with RAMPAGE software^28^. Crystal parameters and refinement statistics are listed in Supplementary Table S1.

### SPR analysis

The interaction between RamR and each substrate was analyzed by SPR spectroscopy with a Biacore T200 biosensor instrument (GE Healthcare). RamR was immobilized onto flow cells in a CM5 sensor chip using an amine-coupling method. Binding analyses were carried out at 25 °C and a flow rate of 30 µl/min. Cholic and chenodeoxycholic acids were passed over the RamR at several concentrations, as indicated. An empty flow-cell lacking immobilized protein was used as a reference. The inhibitory effects of cholic, chenodeoxycholic and deoxycholic acids on the interaction between RamR and P_*ramA*_ were also analyzed by SPR spectroscopy. A 3′-biotinylated 100-bp DNA fragment of the *ramR–ramA* intergenic region, as well as a control 100-bp control fragment of the *gyrB* gene, were each immobilized onto flow cells in a NeutrAvidin (Thermo Scientific)-coated CM5 sensor chip (GE Healthcare). Analyses were performed at 25 °C and at a flow rate of 30 µl/min. The purified RamR protein was diluted in running buffer (10 mM HEPES, pH7.4, 150 mM NaCl, 1 mM EDTA, 0.05% v/v surfactant P20), incubated with 1.0–500 µM of cholic, chenodeoxycholic or deoxycholic acids, then injected onto the sensor surface in two replicates for 2 min. Dissociation was also recorded for 2 min. Calculations of affinity constants were performed using BIAevaluation software (GE Healthcare).

### Accession codes

Protein Data Bank: coordinates have been deposited under accession codes 6IE8 and 6IE9 for the RamR structures co-crystallized with cholic and chenodeoxycholic acids, respectively.

## Electronic supplementary material

Supplementary Information

## References

[1] Prieto AI, Ramos-Morales F, Casadesus J (2004). Bile-induced DNA damage in *Salmonella enterica*. Genetics.

[2] Hernandez SB, Cota I, Ducret A, Aussel L, Casadesus J (2012). Adaptation and preadaptation of *Salmonella enterica* to Bile. PLoS Genet.

[3] Prouty AM, Van Velkinburgh JC, Gunn JS (2002). *Salmonella enterica* serovar typhimurium resistance to bile: identification and characterization of the *tolQRA* cluster. J Bacteriol.

[4] Begley M, Sleator RD, Gahan CG, Hill C (2005). Contribution of three bile-associated loci, *bsh*, *pva*, and *btlB*, to gastrointestinal persistence and bile tolerance of *Listeria monocytogenes*. Infect Immun.

[5] Thanassi DG, Cheng LW, Nikaido H (1997). Active efflux of bile salts by *Escherichia coli*. J Bacteriol.

[6] Gunn JS (2000). Mechanisms of bacterial resistance and response to bile. Microbes Infect.

[7] Nishino K, Latifi T, Groisman EA (2006). Virulence and drug resistance roles of multidrug efflux systems of *Salmonella enterica* serovar Typhimurium. Mol Microbiol.

[8] Prouty AM, Brodsky IE, Falkow S, Gunn JS (2004). Bile-salt-mediated induction of antimicrobial and bile resistance in *Salmonella typhimurium*. Microbiology.

[9] Nikaido E, Yamaguchi A, Nishino K (2008). AcrAB multidrug efflux pump regulation in *Salmonella enterica* serovar Typhimurium by RamA in response to environmental signals. J Biol Chem.

[10] Baucheron S (2014). Bile-mediated activation of the *acrAB* and *tolC* multidrug efflux genes occurs mainly through transcriptional derepression of *ramA* in *Salmonella enterica* serovar Typhimurium. J Antimicrob Chemother.

[11] Abouzeed YM, Baucheron S, Cloeckaert A (2008). *ramR* mutations involved in efflux-mediated multidrug resistance in *Salmonella enterica* serovar Typhimurium. Antimicrob Agents Chemother.

[12] Yamasaki S (2013). The crystal structure of multidrug-resistance regulator RamR with multiple drugs. Nat Commun.

[13] Hofmann AF, Hagey LR, Krasowski MD (2010). Bile salts of vertebrates: structural variation and possible evolutionary significance. J Lipid Res.

[14] Baucheron S (2012). Binding of the RamR repressor to wild-type and mutated promoters of the RamA gene involved in efflux-mediated multidrug resistance in *Salmonella enterica* serovar Typhimurium. Antimicrob Agents Chemother.

[15] Lacroix FJ (1996). *Salmonella typhimurium acrB*-like gene: identification and role in resistance to biliary salts and detergents and in murine infection. FEMS Microbiol Lett.

[16] Baucheron S, Mouline C, Praud K, Chaslus-Dancla E, Cloeckaert A (2005). TolC but not AcrB is essential for multidrug-resistant *Salmonella enterica* serotype Typhimurium colonization of chicks. J Antimicrob Chemother.

[17] Buckley AM (2006). The AcrAB-TolC efflux system of *Salmonella enterica* serovar Typhimurium plays a role in pathogenesis. Cell Microbiol.

[18] Lei HT (2011). Crystal structures of CmeR-bile acid complexes from *Campylobacter jejuni*. Protein Sci.

[19] Gu R (2007). Crystal structure of the transcriptional regulator CmeR from *Campylobacter jejuni*. J Mol Biol.

[20] Li XZ, Plesiat P, Nikaido H (2015). The challenge of efflux-mediated antibiotic resistance in Gram-negative bacteria. Clin Microbiol Rev.

[21] Fields PI, Swanson RV, Haidaris CG, Heffron F (1986). Mutants of *Salmonella typhimurium* that cannot survive within the macrophage are avirulent. Proc Natl Acad Sci USA.

[22] Giraud E, Baucheron S, Virlogeux-Payant I, Nishino K, Cloeckaert A (2013). Effects of natural mutations in the *ramRA* locus on invasiveness of epidemic fluoroquinolone-resistant *Salmonella enterica* serovar Typhimurium isolates. J Infect Dis.

[23] Otwinowski Z, Minor W (1997). Processing of X-ray diffraction data collected in oscillation mode. Methods Enzymol..

[24] Vagin A, Teplyakov A (1997). MOLREP: an automated program for molecular replacement. J. Appl. Cryst..

[25] Brunger AT (1998). Crystallography & NMR System (CNS), A new software suite for macromolecular structure determination. Acta Cryst. D.

[26] Murshudov GN, Vagin AA, Dodson EJ (1997). Refinement of macromolecular structures by the maximum-likelihood method. Acta Cryst. D.

[27] Emsley P, Cowtan K (2004). Coot: model-building tools for molecular graphics. Acta Cryst. D.

[28] Lovell SC (2002). Structure validation by Calpha geometry: phi,psi and Cbeta deviation. Proteins: Structure, Function & Genetics..

