# Supplementary material for: Crystal structure of the multidrug resistance regulator RamR complexed with bile acids
Source: Sci Rep. 2019 Jan 17;9:177. doi: 10.1038/s41598-018-36025-8 (PMC6336783; doi:10.1038/s41598-018-36025-8)
Supplement: Supplementary file 1 — Supplementary Information [file 41598_2018_36025_MOESM1_ESM.doc]

Supplementary Information

Crystal structure of the multidrug resistance regulator RamR complexed with bile acids

Suguru Yamasaki^1^, Ryosuke Nakashima^2^, Keisuke Sakurai^2^, Sylvie Baucheron^3,4^, Etienne Giraud^3,4^, Benoît Doublet^3,4^, Axel Cloeckaert^3,4^ and ^‡^Kunihiko Nishino^1,2^

*^1^Graduate School of Pharmaceutical Sciences, Osaka University, 1-6 Yamadaoka, Suita 565-0871, Osaka Japan. ^2^Institute of Scientific and Industrial Research, Osaka University, 8-1 Mihogaoka, Ibaraki, Osaka 567-0047, Japan. ^3^INRA, UMR1282 Infectiologie et Santé Publique, F-37000 Tours, France. ^4^Université François Rabelais de Tours, UMR1282 Infectiologie et Santé Publique, F-37000 Tours France.*

*^‡^Correspondence should be addressed to K.N. (nishino@sanken.osaka-u.ac.jp).*

| **Supplementary Table S1.** RamR-bile acid complex data collection and refinement statistics | | |
| --- | --- | --- |
| Crystal | RamR + Cholic acid | RamR + Chenodeoxycholic acid |
| **Data Collection** |  |  |
| Light source | SPring8 BL44XU | SPring8 BL44XU |
| Space group | *C* 2 | *C* 2 |
| Wavelength (Å) | 0.9000 | 0.9000 |
| Cell dimensions |  |  |
| *a* (Å) | 87.2 | 87.4 |
| *b* (Å) | 53.6 | 53.6 |
| *c* (Å) | 43.9 | 43.8 |
| *α* (°) | 90.0 | 90.0 |
| *β* (°) | 93.2 | 93.2 |
| *γ* (°) | 90.0 | 90.0 |
| Resolution (Å)* | 50-2.00 (2.03-2.00) | 50-1.78 (1.81-1.78) |
| *R*_merge_ | 5.1(46.9) | 4.0(39.2) |
| CC_1/2_ | (0.949) | (0.952) |
| *I*/σ*I* | 37.8(4.6) | 51.9(5.8) |
| Completeness (%) | 99.1(99.3) | 97.9(97.4) |
| Redundancy | 7.6(7.8) | 7.7(7.8) |
| **Refinement** |  |  |
| Resolution (Å) | 50.0-2.00 | 50.0-1.78 |
| No. reflections | 104,077 | 145,460 |
| *R*_work_/*R*_free_ | 18.8/23.2 | 19.6/25.3 |
| No. atoms |  |  |
| Protein | 1,487 | 1,487 |
| Ligand/ion | 34 | 33 |
| Water | 24 | 36 |
| B-factors |  |  |
| Protein | 44.1 | 42.0 |
| Ligand/ion | 39.4 | 34.0 |
| Water | 39.1 | 41.2 |
| R.m.s. deviations |  |  |
| Bond lengths (Å) | 0.02 | 0.02 |
| Bond angles (°) | 1.998 | 2.009 |
| Ramachandran plot (%)^‡^ |  |  |
| favored region | 95.1 | 96.7 |
| allowed region | 4.4 | 2.7 |
| *The highest resolution shell is shown in parenthesis.  ^‡^Calculated using *RAMPAG* | |  |

| **Supplementary Table S2.** Differences in the interaction of amino acid residues with each compound | | | | | | | |
| --- | --- | --- | --- | --- | --- | --- | --- |
| **R6G** | **EtBr** | **DEQ** | **BER** | **CRY** | **CA** | **CDCA** |  |
| Y59 |  | Y59 |  | Y59 | Y59 | Y59 |  |
| K63 | K63 | K63 | K63 | K63 |  |  |  |
| L66 |  | L66 | L66 | L66 | L66 | L66 |  |
|  |  | C67 |  | C67 |  |  |  |
| M70 |  | M70 |  | M70 | M70 | M70 |  |
| T85 | T85 | T85 | T85 | T85 | T85 | T85 |  |
| I88 | I88 | I88 |  | I88 | I88 | I88 |  |
|  |  |  |  | S91 |  |  |  |
| Y92 | Y92 | Y92 | Y92 | Y92 | Y92 | Y92 |  |
|  |  | H103 |  |  |  |  |  |
| I106 |  | I106 | I106 | I106 |  |  |  |
|  |  | R107 |  |  |  |  |  |
| A110 |  | A110 | A110 | A110 | A110 | A110 |  |
|  |  | V111 |  |  |  |  |  |
|  |  |  |  | F127 |  |  |  |
| L130 | L130 | L130 |  | L130 |  |  |  |
|  | L133 | L133 |  |  |  |  |  |
| C134 | C134 | C134 | C134 | C134 |  |  |  |
| H135 |  |  |  |  |  |  |  |
|  | S137 | S137 |  |  | S137 | S137 |  |
| V138 | V138 | V138 |  | V138 |  |  |  |
|  |  |  |  |  | L139 | L139 |  |
|  |  |  |  |  | M140 | M140 |  |
| F142 |  |  |  |  |  |  |  |
| R148 |  |  | R148 |  |  |  |  |
| G151 |  | G151 | G151 |  |  |  |  |
| D152 | D152 | D152 | D152 | D152 | D152 | D152 |  |
|  |  | G153 |  |  |  |  |  |
| F155 | F155 | F155 | F155 | F155 | F155 | F155 |  |
| L156 |  | L156 |  | L156 | L156 | L156 |  |
|  |  | E160 |  |  |  |  | 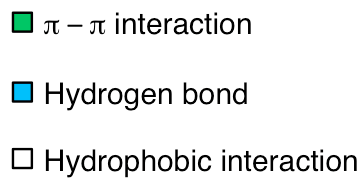 |
| M184 | M184 |  | M184 |  |  |  |  |
|  | W185 |  | W185 |  |  |  |  |
|  | L188 |  | L188 | L188 |  |  |  |
|  | T189 |  | T189 |  |  |  |  |
| BER, berberine; CA, cholic acid; CDCA, chenodeoxycholic acid; CRY, crystal violet; DEQ, dequalinium; R6G, rhodamine 6G; EtBr, ethidium bromide. | | | | | | | |
|

| **Supplementary Table S3**. Strains and plasmids used in this study | | | |
| --- | --- | --- | --- |
| **Strain or plasmid** | **Original name** | **Characteristics** | **Reference or source** |
| **Strains** |  |  |  |
| ***Salmonella* strains** |  |  |  |
| Wild-type | ATCC14028 | *Salmonella enterica* serovar Typhimurium wild-type | 21 |
| Δ*ramR* | Δ*ramR* | Δ*ramR* | 22 |
| ***Escherichia coli* strains** |  |  |  |
| C41 (DE3) |  | F^−^ *ompT* hsdSB(rB^−^ mB^−^) *gal dcm* (DE3) | Lucigen Corporation |
| **Plasmids** |  |  |  |
| pETDuet-1 |  | Vector, Amp^R^ | Novagen |
| pETDuet-1 *ramR* |  | *ramR* gene cloned into pETDuet-1, Amp^R^ | 12 |

| **Supplementary Table S4**. Primers used in this study | |
| --- | --- |
| **Primer** |  |
| **For qRT-PCR** |  |
| *gmk*-f | TTGGCAGGGAGGCGTTT |
| *gmk*-r | GCGCGAAGTGCCGTAGTAAT |
| *gyrB*-f | TCTCCTCACAGACCAAAGATAAGCT |
| *gyrB*-r | CGCTCAGCAGTTCGTTCATC |
| *rrs*-f | CCAGCAGCCGCGGTAAT |
| *rrs*-r | TTTACGCCCAGTAATTCCGATT |
| *ramA*-f | GCGTGAACGGAAGCTAAAAC |
| *ramA*-r | GGCCATGCTTTTCTTTACGA |
| **For SPR assay** |  |
| *ramRA*_PR_100F | CTTTGAAAAGTACCTTGACGGCG |
| *ramRA*_PR_100R | CAATATGCTTTTCTACCACTTC |

SPR, surface plasmon resonance.
